# Upward Ingratiation Outside the Workplace and Supervisor’s Human Resource Decisions: Moderating Effect of Zhongyong Thinking

**DOI:** 10.3389/fpsyg.2021.636756

**Published:** 2021-05-26

**Authors:** Hui Sun, Haibing Guo, Kai Wang, Ling Sun, Lu Wang

**Affiliations:** ^1^School of Economics and Management, China University of Mining and Technology, Xuzhou, China; ^2^School of Business, Jiangsu Ocean University, Lianyungang, China; ^3^School of Science, Jiangsu Ocean University, Lianyungang, China; ^4^School of Environmental Engineering, Xuzhou Institute of Technology, Xuzhou, China

**Keywords:** upward ingratiation outside the workplace, leader-member exchange, Zhongyong thinking, chance of promotion, bonus allocation, participation in the decision-making

## Abstract

Ingratiation is a common strategy for subordinates to deal with their supervisors in eastern and western societies. Based on the theory of impression management, this study focuses on the impact of upward ingratiation outside the workplace on supervisor’s human resource (HR) decisions in the Chinese context and the mechanism behind this impact. The data were collected from 252 supervisor-subordinate dyads in four manufacturing firms. The results demonstrate the following: first, supervisors hold a more favorable view of upward ingratiation outside the workplace; second, upward ingratiation outside the workplace has a positive effect on the supervisor’s chance of promotion and bonus allocation decisions, and leader-member exchange (LMX) plays a mediation role in this influence; third, Zhongyong thinking (ZYT) moderates the relationship between LMX and supervisor’s chance of promotion and bonus allocation decisions; and finally, ZYT moderates the indirect effect of ingratiation behavior outside the workplace on supervisor’s chance of promotion and bonus allocation decisions through LMX, and the mediated relationship is weakened when a supervisor has a higher level of ZYT. This is one of the first empirical studies, which examines the validity of subordinate’s upward ingratiation outside the workplace from the perspective of supervisor’s ZYT. This study plays an important role in highlighting the effect of ZYT on the ingratiation behavior.

## Introduction

In the past few decades, one of the most important changes in the field of human resource (HR) management (HRM) has been the increasing responsibility of supervisors in making HR decisions (Purcell and Hutchinson, 2007; Brewster et al., 2015). Supervisor’s HR decisions have a substantial impact on the subordinates they manage (Clarke et al., 2019). Although supervisor’s HR decisions are rooted in a complex and dynamic social context (Wayne et al., 1997), there is a possibility that these decisions are biased (Clarke et al., 2019). Therefore, subordinates can take some initiative actions to influence the key HR decisions from which they perceive that they could obtain some more (Harris et al., 2007). A large number of literatures have accumulated, showing that subordinates’ particular behaviors (i.e., upward ingratiation behaviors) are associated with supervisor’s HR decisions (Terpstra-Tong and Ralston, 2002; Lee et al., 2017). The impression management studies try to explain how the upward ingratiation behavior affects the HR decisions (Bolino et al., 2008); however, they have not reached an agreement on the relationship between the upward ingratiation and its consequences. Researchers found that the upward ingratiation leads to the higher compensation (Judge and Bretz, 1994), career advancement (Sibunruang et al., 2016), better job performance and promotion (Wu et al., 2013), and enhanced trust from their managers (Su, 2010). However, some studies have investigated that the ingratiating behavior has no significant influence on salary (Aryee et al., 1996), performance, and promotion (Rao et al., 1995).

The above-mentioned inconsistent results can be attributed to three aspects: (1) the effective ingratiation should be linked with a specific scenario. Wayne et al. (1997) believed that insufficient attention was paid to the situational factors of the ingratiation and its effects. Most of the existing studies focus on the ingratiation behavior in the workplace (Liu et al., 2015); however, very few of them focus on the ingratiation behavior outside the workplace. In many countries, such as China, the upward ingratiation behavior prevails outside the workplace, including presenting gifts and greetings on holidays and paying attention to the personal needs of supervisors (Mayfair, 1994). Huang and Hu (2004) showed that Chinese pay more attention to human sympathy, relationship, and face, most of which are established and developed in informal situations. In China, most of the communication and cooperation between subordinates and supervisors are conducted at the end of work or after the work, which are conducive to develop high-quality work relationships (Xu and Liang, 2008). Therefore, in China, the really effective ingratiation behavior is likely to happen outside the workplace. (2) The existing studies focus on the report of subordinates while ignoring the supervisors’ perception of ingratiation (e.g., Rao et al., 1995; Aryee et al., 1996). This omission is curiously considered that the supervisors are the key decision-makers in their subordinates’ performance rating and promotion (Hoobler et al., 2009). (3) Most of the existing studies have examined the direct relationship between ingratiation and subordinates’ career outcome, failing to establish a theoretical framework to interpret “under what conditions the ingratiation is favorable or harmful to the performance outcomes” (Judge and Bretz, 1994; Wayne et al., 1997). To solve this problem, it is important to put forward a new classification framework, to clarify the complexity in the ingratiation process, and to consider the potential boundary condition and mediators related to the complicated processes.

The first objective of this study is to investigate the influence of the ingratiation outside the workplace on supervisor’s HR decisions in the Chinese organizations on the basis of dividing the upward ingratiation into ingratiation in the workplace and ingratiation outside the workplace. Liu et al. (2015) distinguished the ingratiation behavior between inside and outside the workplace, and explored the impact of ingratiation outside the workplace on subordinates’ promotion. However, current studies do not concentrate on the impact of ingratiation outside the workplace on the other HR decisions of supervisors, such as opportunities to participation in decision-making and bonus allocation. Although some authors have proposed that relationships tend to go further than just the workplace setting (Bauer and Ergoden, 2015), there is no research on the ingratiation behavior outside the workplace in foreign countries. Therefore, it is expected that through the study of this article, the researchers can shift their focuses to ingratiation outside the workplace.

The second objective of this study is to use leader-member exchange (LMX) as an intermediary mechanism, through which the upward ingratiation may affect the HR decisions of supervisors. Supervisor’s liking has been found to be positively correlated with supervisor’s reward behavior (Ferris, 2014; Helmut, 2018). Liu et al. (2015) used supervisor’s liking as a mediation variable to explain the relationship between subordinate’s ingratiation behavior and supervisor’s promotion decision. This draws upon social exchange theory (Blau, 1964), assuming that supervisors will make more favorable responses in their HR decisions to the subordinates who have relationship with them. However, HR decision-making depends more on the decision-maker’s judgment on subordinates and supervisor’s liking may be unstable over a long period of time. Weng and Chang (2015) reckoned LMX to be a relatively stable concept in the long run. LMX has been considered as a possible mediator between upward ingratiation and HR decisions (Nahrgang and Seo, 2015). Therefore, this study considers LMX more suitable for explaining the influence of upward ingratiation on supervisor’s HR decisions.

The third objective of this study is to investigate the moderating effects of Zhongyong thinking (ZYT) among the ingratiation behavior, LMX, and supervisor’s HR decisions. Ingratiation behavior is the interpersonal and social behavior involving both the supervisors and the subordinates. Therefore, we should not only examine those who implement the ingratiation in isolation but also consider more about the subjective feelings and personal characteristics of supervisors. Supervisors with different traits have different recognitions and preferences for the ingratiation behavior of their subordinates. Culture is perceived as having a profound and permanent influence on the way of thinking and behavior of individuals (Nisbett and Miyamoto, 2005; Miyamoto et al., 2006; Nathan and Lee, 2013). It is manifested by the study of Nisbett et al. (2000) that Western and Chinese cultures have developed diverse thoughts because of their differences in historical development. Western culture is inclined to adopt analytical thinking and logical concepts. In contrast, Chinese culture prefers dialectical thinking and harmony between man and nature. In other words, holism characteristics, contradiction acceptance, and harmony-centered thoughts are always popular in China, which is often termed as ZYT (Yang, 2009). ZYT (the Doctrine of the Mean) is one of the central thinking modes for the Chinese people (Chang and Yang, 2014). As an indigenous construction reflecting the traditional Confucian culture (Chou et al., 2014; Pan and Sun, 2018), ZYT is a complex cognitive thinking about how Chinese view things, people, and environment (Pierce and Aguinis, 2013; Chou et al., 2014). Specifically, ZYT refers to the thinking mode of how to integrate external conditions with internal needs from multiple perspectives and take practical actions under specific circumstances (Wu and Lin, 2005), and it analyzes what are the good and bad parts in each of the two opposite opinions and then combines the good parts while casting off the bad parts of both opposite opinions (Li, 2018). Individuals with higher ZYT can combine external conditions to adjust their views and integrate them into the thinking of others (Wei et al., 2020). ZYT emphasizes that individuals need to examine the environment from multiple perspectives before action and solve the problems with appropriate methods for achieving goals harmoniously. It is the most prominent cognitive attitude that is adopted in social interaction in Oriental culture, which affects the Chinese behavior and working principles (Chou et al., 2014). Chen et al. (2010) applied ZYT to the leadership theory and analyzed how the leader’s ZYT influenced the organization performance. However, the supervisor’s ZYT was rarely involved in the research of the ingratiation behavior. ZYT is included in the current model because as an important moderator, it can explain the differences in individual behaviors in the same situation (He, 2009).

**Figure 1:**
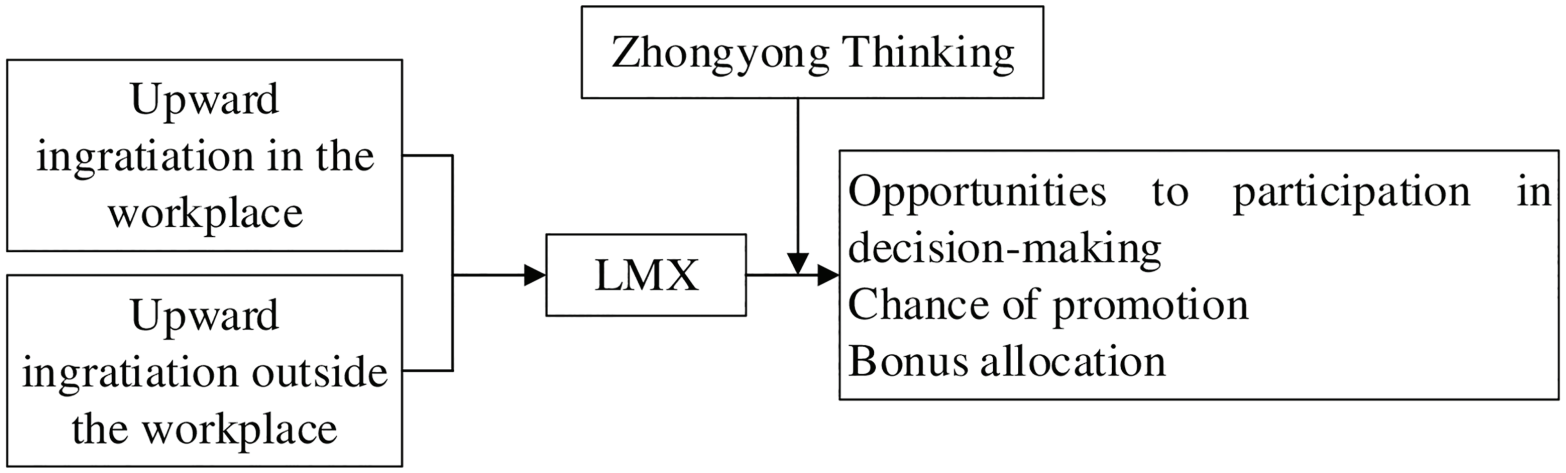
Research framework.

We applied the theory of impression management to verify a comprehensive model of the social effect process that concerns the relationships of ingratiation outside the workplace with supervisor’s HR decisions by focusing on the moderating role of LMX and the mediating role of supervisor’s ZYT. The conceptual model of this study is illustrated in Figure 1. The possible contributions of this study mainly include the following aspects: first, this study expands the research context of the impression management strategy, introduces a new perspective for the existing ingratitude research to the West, and provides the reference experience for the follow-up related research; second, it helps to reveal some of the complex mechanisms that arise between subordinates and supervisors outside the workplace (i.e., I ingratiate my supervisor outside the workplace. My supervisor will perceive my ingratiation and establish better workplace relationship with me), and to inspect how these mechanisms affect the career outcomes of subordinates in the workplace, which broaden and deepen our understanding of ingratiation; and third, this study is conducive to the HR literature in furthering our understanding of individual (e.g., ingratiation outside the workplace) and contextual (e.g., LMX) factors that impact supervisor’s HR decisions in areas, and it can offer significant implications for the HRM practice.

## Theory and Hypothesis

### Relationship Between Upward Ingratiation Outside the Workplace and Supervisor’s HR Decisions

As an important social influence behavior, the ingratiation has received considerable research attention because it is the most prevailing impression management strategy in the workplace (Rao et al., 1995; Wu et al., 2013). Previous researches have tested the association between the ingratiation and its career outcomes, but the results have been highly inconsistent (Wu et al., 2013). For example, De Clercq et al. (2019) have found that the ingratiation behavior helps subordinates attain higher status in the organization, whereas other studies have revealed that the ingratiation behavior has no significant impact on subordinate’s promotion (Aryee et al., 1996). This makes empirical studies research on the influence mechanism of ingratiation behavior to emerge gradually and adds a lot of contributions to this field (Wayne et al., 1997; Wu et al., 2013). However, an obvious drawback of all these studies is that the researches and measurements of ingratiation behavior are limited to the workplace, and the subordinate’s ingratiation behavior outside the workplace is basically not considered.

The impression management theory suggests that the effectiveness of ingratiation depends on how it is delivered (Goffman, 1959). Compared with the ingratiation in the workplace, the ingratiation outside the workplace is more locally compatible with the Chinese culture. The Chinese likes the human relationship and face mostly shaped and developed in informal situations (Mayfair, 1994). Outside the workplace, subordinates and supervisors socialize on “public affairs” or “private affairs” with some human exchanges, which is more likely to be an advantage to them (Liu et al., 2015). In state-owned enterprises that have an imperfect modern enterprise system and a strong political atmosphere, the ingratiation outside the workplace is more universal and effective (Huang and Hu, 2004).

The impression management proposes that individuals usually try to shape and maintain appropriate impressions in social situations for obtaining positive evaluation (Goffman, 1959). From the Chinese culture perspective, in order to develop their own relationship network, supervisors are generally willing to accept the upward ingratiation. Subordinates seek to ingratiate in attempt to win the recognition of supervisors and tend to ingratiate outside the workplace showing the same emotional orientation to supervisors. At this point, based on the principle of “reciprocity,” once supervisors perceive to the ingratiation, they will feel obligated to use their own powers and resources to reciprocate after experiencing the subordinates’ kindness, such as offering them more bonus allocation, chance of promotion, and awarding greater opportunities to participate in decision-making. Therefore, we proposed the following hypotheses:

H1a: Controlling for ingratiation in the workplace, the upward ingratiation outside the workplace has a positive effect on participation in decision-making.

H1b: Controlling for ingratiation in the workplace, the upward ingratiation outside the workplace has a positive effect on chance of promotion.

H1c: Controlling for ingratiation in the workplace, the upward ingratiation outside the workplace has a positive effect on bonus allocation.

### The Mediation Effect of LMX

The impression management theory assumes that people accumulate their emotion toward a person continuously (Goffman, 1959). It considers that a subordinate’s ingratiation behavior can cause a change in the supervisor’s attitude (Goffman, 1959). LMX and career outcomes are the appropriate outcomes in the impression management theory framework because they reflect the results of continuous interpersonal interaction and assessment processes (Weng and Chang, 2015). LMX relation is grounded in the social exchange theory (Blau, 1964). The theory points out that the purpose of interpersonal relationship ties is to gain greater reciprocity in the future. The subordinates who wish to benefit from their supervisors will adopt the ingratiation behavior and take the initiative to establish an exchange relationship with their supervisors (Treadway et al., 2007). Although the establishment of this kind of relationship is the result of subordinates’ ingratiation behavior, positive emotional connection bonding will still be produced during this interaction (Weng and Chang, 2015). When subordinates engage in ingratiation outside the workplace, their supervisors will perceive and respond to the subordinates’ ingratiation behavior (Treadway et al., 2007; Liu et al., 2015). If a supervisor is in favor of proper upward ingratiation, and the subordinate also selects an appropriate setting and means of implementing upward ingratiation, under the impression management framework, the supervisor will have a sense of liking to the subordinate (Liu et al., 2015; Helmut, 2018). From the dynamic perspective of impression management, subordinates are likely to perceive the supervisors’ recognition, which certainly encourages the ingratiation behavior, i.e., in the dyadic interactions between supervisors and subordinates, the ingratiation behavior adopted by the subordinates successfully affects the LMX relationship.

Building high-quality LMX in the workplace is only a way through which subordinates can adopt some ingratiation behavior to achieve career success, but it is not the ultimate goal (Zhang and Jia, 2016). The impression management theory considers that the successful implementation of the ingratiation can make the target feel good, which leads the target produce positive reciprocations to the participant (Goffman, 1959). Earlier studies have shown that subordinates with high-quality LMX relationships with their supervisors gain a number of advantages and benefits such as better opportunities (Usman et al., 2021), more recognition (Herman and Mitchell, 2010), formal and informal rewards (Wayne et al., 2004; Ilies et al., 2007), career development (Liden et al., 2000; Breland et al., 2007; Naseer et al., 2016), easier access to different resources (Tummers and Knies, 2013), and performance rating (Harris et al., 2009; McLarty et al., 2021). Since the supervisor has the decision-making power of important resources, including decision-making of work assignment, performance evaluation, or promotion decision, the supervisor is more inclined to give good resources or opportunities to those who have high-quality LMX. Therefore, this study proposes the following hypotheses:

H2a: LMX mediates the relationship between upward ingratiation outside the workplace and participation in decision-making.

H2b: LMX mediates the relationship between upward ingratiation outside the workplace and chance of promotion.

H2c: LMX mediates the relationship between upward ingratiation outside the workplace and bonus allocation.

### Moderating Effect of Zhongyong Thinking

The impression management theory holds that the main action motivation is to avoid being negatively evaluated (Bozeman and Kacmar, 1997; Chiu, 2000; Jain, 2012). Wu and Lin (2005) proposed three characteristics of the ZYT: multiple thinking, integration, and harmoniousness. When making the three types of HR decisions, namely, decision-making participation, chance of promotion, and bonus allocation, the supervisor usually examines the situation and deflects negative evaluation from others (Treadway et al., 2007). If the supervisor uses an authority to take special care of his/her close subordinates, he/she may worry that this will affect his/her image of “justice” and “selflessness”; however, if the supervisor does not do that way, he/she needs to worry about the formation of his/her personal circle. Facing the above-mentioned contradictions, first, supervisors with higher ZYT tend to consider multiple dimensions in terms of time, space, and roles, and estimate LMX from a long-term perspective (Chou et al., 2014), which implies that they are unlikely to consider only LMX when making HR decisions. Second, the connotation of integration in ZYT motivates the supervisors to integrate a variety of resources, for instance, the opinions of subordinates and others (Wu and Lin, 2005). In that case, after repeated thinking, integration, and optimization, the supervisors finally find the solution that can satisfy most of the people (Yang, 2009). Third, supervisors with higher ZYT seek to pursue the aim that their ultimate decision can maintain interpersonal harmony (Yang, 2009; Spencer-Oatey, 2013). When they are closer to some subordinates, they should care more about how to make HR decisions positively, rather than whether to do or not (Wu and Lin, 2005; Yang, 2009). When making the three types of HR decisions, supervisors with higher ZYT will first take into account the overall interpersonal harmony within the organization or department, before prioritizing insiders out of personal preference. In general, supervisors with higher ZYT can think the issue from many angles, integrate opinions, and handle affairs harmoniously, thus weaken the impact of LMX on their HR decisions. Based on this view, this study proposes the following hypotheses:

H3a: ZYT moderates the relationship between LMX and participation in decision-making; the positive relationship between LMX and participation in decision-making is weaker among supervisors with higher ZYT than among those with lower ZYT.

H3b: ZYT moderates the relationship between LMX and chance of promotion; the positive relationship between LMX and chance of promotion is weaker among supervisors with higher ZYT than among those with lower ZYT.

H3c: ZYT moderates the relationship between LMX and bonus allocation; the positive relationship between LMX and bonus allocation is weaker among supervisors with higher ZYT than among those with lower ZYT.

In addition, given the potential role of ZYT in the effect of LMX on supervisor’s HR decisions, it is likely that second-stage moderation exists in the mediated relationship between ingratiation outside the workplace and supervisor’s HR decisions *via* LMX (Edwards and Lambert, 2007), though the ingratiation outside the workplace might lead to improved LMX which may have different effects on supervisor with different levels of ZYT. As suggested by Zhang et al. (2001), ZYT is highly principled and it emphasizes self-restraint as well as impartiality. The effectiveness of the ingratiation behavior depends on whether subordinates can effectively conceal the egoistical purpose behind the behavior (Wu et al., 2013). Supervisors with higher ZYT have strong principles and they insist on executing what they think is right and do not favor one side over another. This principle enables a supervisor with higher ZYT to maintain a balanced attitude in the face of subordinates’ compliments. This kind of balanced attitude helps to clarify the motivations of other people. In addition, this principle can lead supervisors with higher ZYT to evaluate their subordinates’ abilities impartially. In this case, it is no longer meaningful for subordinates to engage in the ingratiation. On the contrary, the supervisor with lower ZYT is less sensitive to the external environment, he/she usually does not consider the impact of his/her behavior on the whole situation and cannot flexibly adjust his/her behavior according to the situation (He, 2009; Chen et al., 2010, 2017), and he/she tends to take advantage of his/her own authority to take care of the subordinates with high-quality LMX relationship. In this case, the proper ingratiation of subordinates can play their due role. Therefore, we predicted that:

H4a: ZYT moderates the indirect effect of ingratiation behavior outside the workplace on participation in decision-making through LMX, such that the mediated relationship is weakened when a supervisor has a higher level of ZYT.

H4b: ZYT moderates the indirect effect of ingratiation behavior outside the workplace on chance of promotion through LMX, such that the mediated relationship is weakened when a supervisor has a higher level of ZYT.

H4c: ZYT moderates the indirect effect of ingratiation behavior outside the workplace on bonus allocation through LMX, such that the mediated relationship is weakened when a supervisor has a higher level of ZYT.

## Materials and Methods

### Participants and Procedures

The data in this study were collected from four traditional production-oriented enterprises, two were in Xuzhou and Lianyungang both situated in Jiangsu Province, and the other two were in Shanghai. Since the questionnaire involved sensitive workplace issues, in order to ensure employees’ active participation, on the one hand, we promised the respondents that we assured their responses confidential, and explained the questionnaire to the respondents solely for ensuring the best HRM practice performance. On the other hand, employees were free to decide whether they participate in the questionnaire, and the financial compensation was provided to the participant employees to improve the response rate. All participants read the participant information statement and furnished the online informed consent before the questionnaire survey. The questionnaires were coded before handed out, and the HRM department of the companies involved helped record the identity numbers and the names of the interviewees for matching supervisor-subordinate dyads (i.e., one supervisor rated only one subordinate). During the work hours, the respondents acquired the questionnaires face-to-face from information collectors who were trained and led by one of the authors in a conference room. Besides, for avoiding the common method bias problem as much as possible, this study employed a time-lagged analysis (Podsakoff et al., 2003). At ‑the initial time point (i.e., time point 1), the respondents of the questionnaire survey were mainly composed of subordinates. The survey primarily includes the demographic characteristics of subordinates (e.g., company, gender, age, and tenure with the supervisor), subordinates’ ingratiation in and outside the workplace, and LMX of subordinates’ evaluation. With the help of the HR department of the company involved, a list of randomly selected 442 subordinates and their corresponding 442 supervisors was compiled, and 322 valid questionnaires were retrieved (i.e., effective recovery rate of 72.9%). The respondents put their completed questionnaires in sealed envelopes and placed them into a box set up in the HR department. One month later (i.e., time point 2), the respondents of the questionnaire covered the supervisors who have been matched with the subordinate who completes the questionnaire for the first time. The contents of the questionnaire included the supervisors’ demographics (e.g., age and gender), the supervisors’ ZYT, and HR decisions to the paired subordinates, such as opportunities of participation in decision-making, chance of promotion, and bonus allocation. Finally, 252 valid and matched data were obtained by matching the data of subordinates and supervisors (i.e., effective recovery rate of 78.3%).

The sample involved 252 subordinates, 58.6% of whom were males and mainly aged below 25 and 26–35 years (i.e., 30.2 and 44.1%, respectively). Among the 252 supervisors, 60.7% were males whose age mainly ranged between 26 and 35 years and 36 and 45 years (i.e., 29.4 and 38.5%, respectively). The employment duration of supervisors and subordinates primarily fell between 1 and 5 years and 6 and 10 years (i.e., 43.7 and 31.7%, respectively).

### Measures

#### Upward Ingratiation in the Workplace

This study slightly modified the “ingratiation in the workplace” scale, which was proposed by Westphal and Bednar (2008). For example, in the original questionnaire, this study amended the statement, “I would like to express my appreciation for the way my supervisor handles his work” to “In the workplace, I would like to express my appreciation for the way my supervisor handles his work.” In this study, the Cronbach’s alpha coefficient of this scale was 0.87.

#### Upward Ingratiation Outside the Workplace

A 7-item scale, which was developed by Liu et al. (2015), was used to assess the ingratiation by subordinates outside the workplace. A sample item was “In my spare time, I often gauge the potential needs of my supervisors and try my best to satisfy them in order to win his appreciation.” The Cronbach’s alpha coefficient was 0.92.

#### Leader-Member Exchange

A 7-item scale, developed by Graen and Uhl-Bien (1995), was used to measure LMX. A sample item was “My immediate supervisor understands my problems and needs.” The Cronbach’s alpha coefficient was 0.92.

#### Zhongyong Thinking

A 13-item scale, developed by Wu and Lin (2005), was used to measure ZYT. It includes “I am used to thinking about one thing from different perspectives” and “I usually adjust my behavior for overall harmony.” The Cronbach’s alpha coefficient was 0.92.

#### Participation in Decision-Making

A 3-item scale, developed by Xu et al. (2005), was used to measure the participation in decision-making. It includes “I would try my best to give him/her the opportunity to participate in decision-making” and “When making decisions, I would try my best to consider his/her opinions.” The Cronbach’s alpha coefficient was 0.82.

#### Chance of Promotion and Bonus Allocation

Both scales were developed by Law et al. (2000). A 4-item scale is used to measure the chance of promotion, such as “I would try my best to promote him/her.” The Cronbach’s alpha was coefficient 0.85. A 3-item scale is used to measure bonus allocation such as “I give very reasonable bonus to him/her.” The Cronbach’s alpha coefficient was 0.75.

#### Control Variable

Based on the previous findings (e.g., Judge and Bretz, 1994; Spencer-Oatey, 2013; Liu et al., 2015), we controlled the demographic variables among subordinates (i.e., company, age, gender, and tenure with the supervisor) and supervisors (i.e., age and gender), as well as upward ingratiation in the workplace.

All of the above scales are scored by a 5-point Likert-type scaling method, and it is noticed that 1–5 represent a gradual increase in the degree of conformity.

## Data Analysis and Results

### Confirmatory Factor Analysis and Common Method Bias Control

Given our relatively small sample size, the confirmatory factor analysis (CFA) was carried out to assess the discriminant validity of the seven-factor (namely, upward ingratiation in the workplace, upward ingratiation outside the workplace, LMX, ZYT, participation in decision-making, chance of promotion, and bonus allocation) model using Amos 23.0 software. We evaluated the model fit by using the various indices adopted by Dong et al. (2014). The seven-factor model fits well [*χ*^2^(795) = 1,416.62, *p* ≤ 0.01, comparative fit index (CFI) = 0.91, Tucker-Lewis index (TLI) = 0.90, root mean square error of approximation (RMSEA) = 0.04, standardized root mean square residual (SRMR) = 0.05], and it is better than the alternative models. The results are shown in Table 1. In addition, all factor loadings were greater than 0.5 and significant, and composite reliabilities were all above 0.80. At the same time, convergent and discriminant validity were examined by following the method of Hair et al. (2006). The average variance extracted (AVE) of each variable was above 0.5, and all square roots of these estimates exceeded the correlation between the factors making up each pair. The above-mentioned results show that the seven constructs can be applied in further analyses.

**Table 1:** Results of confirmatory factor analysis for the measures of the variables studied.

Model	*χ*^2^/*df*	CFI	TLI	RMSEA	SRMR
Seven-factor model	1.78	0.91	0.90	0.05	0.04
Six-factor model 1: IOW and IIW combined	2.01	0.89	0.88	0.07	0.06
Six-factor model 2: IIW and ZYT combined	2.23	0.86	0.85	0.09	0.06
Six-factor model 3: ZYT and CP combined	2.12	0.88	0.86	0.08	0.06
Six-factor model 4: PDM and CP combined	2.34	0.85	0.85	0.08	0.06
Six-factor model 5: CP and BA combined	2.07	0.89	0.88	0.09	0.06
Six-factor model 6: LMX and PDM combined	2.85	0.85	0.84	0.11	0.07

LMX, leader-member exchange; ZYT, Zhongyong thinking; IIW, ingratiation in the workplace; IOW, ingratiation outside the workplace; PDM, participating in decision-making; CP, chance of promotion; BA, bonus allocation.

Since all the measurement scales were self-reported, there may be a potential for common method variance (CMV) problems. In order to reduce the impact of CMV on the research results, first, the principles of confidentiality and voluntariness were strictly followed to control the bias in research design as a remedy in the questionnaire survey. Second, the Harman’s single-factor test was used to analyze the common method variation of the data (Carlson and Kacmar, 2000). The results show that the nine factors are extracted from the non-rotating principal component analysis with 69.92% of the total variance, among which the first factor explained 20.94%, indicating that CMV is unlikely to affect the results (Podsakoff et al., 2003).

### Descriptive Statistical Analyses

The correlation coefficient and descriptive statistics of each variable are shown in Table 2.

**Table 2:** Means (M), SDs, and correlations.

Variable	1	2	3	4	5	6	7	8	9	10	11	12	13
1. C	1												
2. SUBG	−0.01	1											
3. SUBA	−0.08	0.04	1										
4. SUPG	−0.01	−0.04	−0.09	1									
5. SUPA	−0.01	−0.44^**^	0.03	−0.15^*^	1								
6. TWS	−0.12	−0.10	0.46^**^	−0.27^*^	0.25^**^	1							
7. IIW	0.03	−0.29^**^	−0.03	−0.15^*^	0.32^**^	0.09	1						
8. IOW	0.09	−0.01	−0.00	−0.28^**^	0.19^**^	0.08	0.34^**^	1					
9. LMX	−0.03	−0.33^**^	0.04	−0.10	0.31^**^	0.12	0.52^**^	0.38^**^	1				
10. ZYT	0.10	0.03	0.06	−0.06	−0.07	−0.05	−0.03	0.03	−0.03	1			
11. PDM	0.08	−0.03	0.12	0.12	0.01	−0.07	−0.03	0.08	0.03	−0.05	1		
12. CP	0.04	−0.17^**^	0.02	−0.16^**^	0.36^**^	0.12	0.18^**^	0.34^**^	0.33^**^	−0.10	0.20^**^	1	
13. BA	0.05	−0.10	0.03	−0.09	−0.03	0.05	0.18^**^	0.32^**^	0.34^**^	0.15^*^	0.07	0.36^**^	1
M	2.56	1.47	1.98	1.61	2.92	2.25	2.75	2.90	3.07	3.15	3.04	3.16	3.10
SD	1.10	0.50	0.81	0.49	0.83	0.83	0.58	0.70	0.70	0.60	0.67	0.70	0.65

*n* = 252. C, company; SUBG, subordinate gender; SUBA, subordinate age; SUPG, supervisor gender; SUPA, supervisor age; TWS, tenure with the supervisor; LMX, leader-member exchange; ZYT, Zhongyong thinking; IIW, ingratiation in the workplace; IOW, ingratiation outside the workplace; PDM, participating in decision-making; CP, chance of promotion; BA, bonus allocation.

* *p* < 0.05;

** *p* ≤ 0.01.

A preliminary assessment of Table 2 indicates that there is a positive relationship between ingratiation outside the workplace and LMX (*r* = 0.38, *p* < 0.01), participation in decision-making (*r* = 0.08, *p* > 0.05), chance of promotion (*r* = 0.34, *p* < 0.01), and bonus allocation (*r* = 0.32, *p* < 0.01).

### Test of Hypotheses

We used Amos 23.0 software to verify the H1a–H1c, H2a–H2c, and H3a–H3c. First, we tested the effect between ingratiation in the workplace and supervisors’ HR decisions. The fit index revealed that the model fits well into the observed covariance structure of the sample [*χ*^2^_(99)_ = 216.79, *p* ≤ 0.01, CFI = 0.93, TLI = 0.92, RMSEA = 0.07, SRMR = 0.04]. The results showed that the ingratiation in the workplace of subordinates had a significant effect on chance of promotion (*β* = 0.21, *p* < 0.01) and bonus allocation (*β* = 0.18, *p* < 0.05), but there is no significant effect on participation in the decision-making (*β* = 0.04, *p* > 0.05). Then, we tested the direct effect between ingratiation outside the workplace and supervisors’ HR decisions while controlling for the ingratiation in the workplace. The results showed that the ingratiation outside the workplace of subordinates had a significant effect on chance of promotion (*β* = 0.43, *p* < 0.01) and bonus allocation (*β* = 0.29, *p* < 0.05), but there is no significant effect on participation in the decision-making (*β* = 0.13, *p* > 0.05). In other words, H1b and H1c were supported, but H1a was not supported. Furthermore, when the ingratiation outside the workplace was added, the results showed that the impact of ingratiation in the workplace on chance of promotion (*β* = 0.03, *p* > 0.05) and bonus allocation (*β* = 0.09, *p* > 0.05) became insignificant. Therefore, it can be said that supervisors hold a more favorable view about ingratiation outside the workplace.

**Figure 2:**
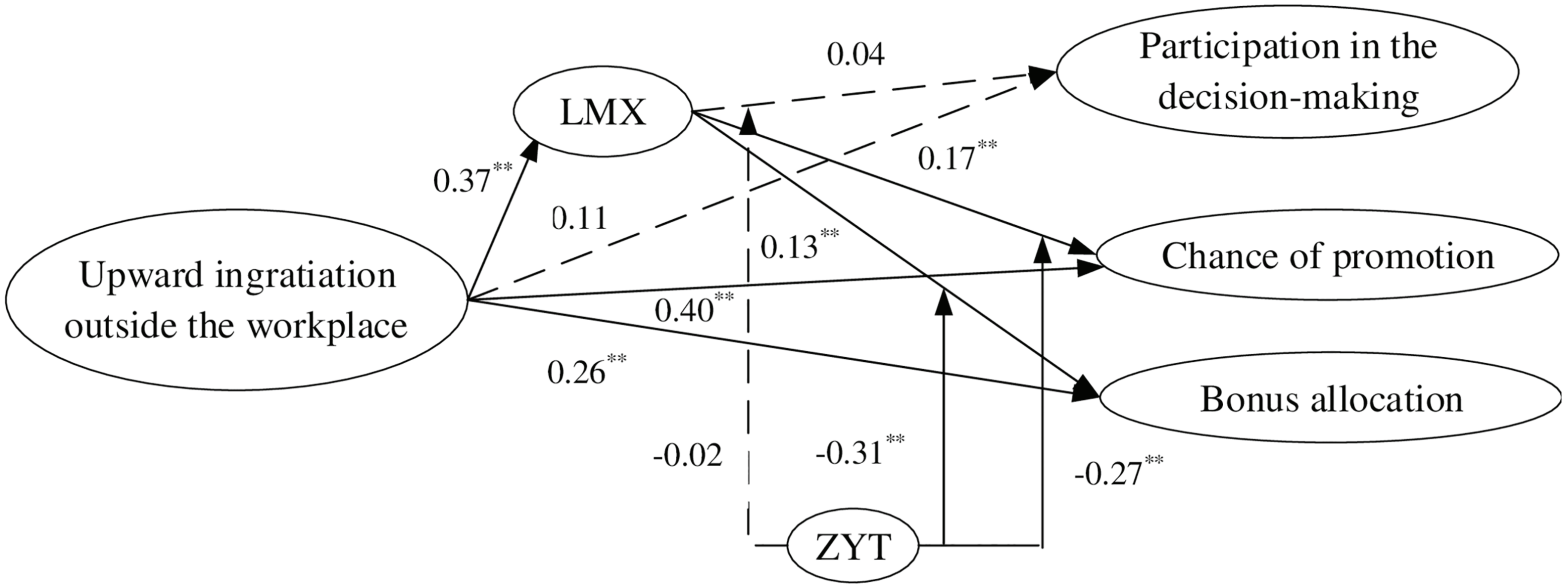
Simplified mediation model. *n* = 252. ^**^Significant at the *p* ≤ 0.01 level.

Second, we verified the mediation effect of LMX between ingratiation outside the workplace and supervisors’ HR decisions. The fit index revealed that the model fits well into the observed covariance structure of the sample [*χ*^2^_(377)_ = 784.46, *p* ≤ 0.01, CFI = 0.92, TLI = 0.90, RMSEA = 0.07, SRMR = 0.05]. As shown in Figure 2, the bonus allocation (*β* = 0.26, *p* < 0.01), chance of promotion (*β* = 0.40, *p* < 0.01), and LMX (*β* = 0.37, *p* < 0.01) were significantly affected by ingratiation outside the workplace. Although bonus allocation (*β* = 0.13, *p* < 0.01) and chance of promotion (*β* = 0.17, *p* < 0.01) were significantly affected by LMX, the effect of LMX on participation in the decision-making was not significant (*β* = 0.04, *p* > 0.05). In other words, LMX played a partial mediation role in the relationship between ingratiation outside the workplace and bonus allocation, as well as in the relationship between ingratiation outside the workplace and chance of promotion, but it did not play a mediation role in the relationship between ingratiation outside the workplace and participation in the decision-making. That is, H2a was not supported, but H2b and H2c were supported.

Third, we verified the hypothesis that the association between ingratiation outside the workplace and supervisors’ HR decisions would be reduced by ZYT and the results are shown in Figure 2. For H3b, the fit index revealed that the model fits well into the observed covariance structure of the sample [*χ*^2^_(274)_ = 406.43, *p* ≤ 0.01, CFI = 0.92, TLI = 0.91, RMSEA = 0.06, SRMR = 0.05], i.e., the interaction of LMX and ZYT on chance of promotion was significant (*β* = −0.27, *p* < 0.01). For H3c, the fit index revealed that the model also fits well into the observed covariance structure of the sample [*χ*^2^_(315)_ = 587.46, *p* ≤ 0.01, CFI = 0.91, TLI = 0.91, RMSEA = 0.06, SRMR = 0.06], i.e., the interaction of LMX and ZYT on bonus allocation was significant (*β* = −0.31, *p* < 0.01).

**Figure 3:**
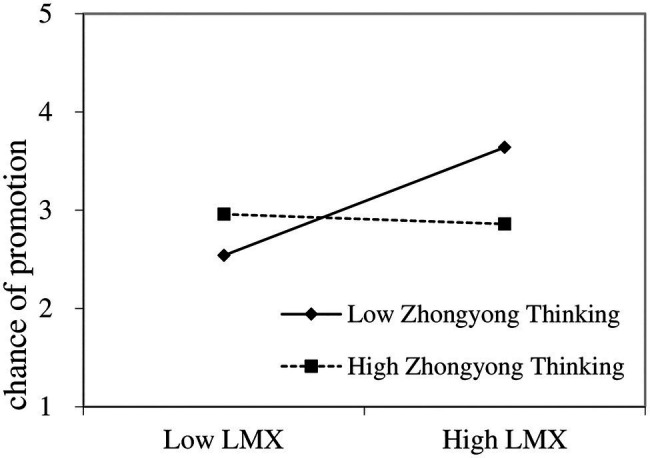
Moderating role of Zhongyong thinking (ZYT) in the relationship between leader-member exchange (LMX) and chance of promotion.

**Figure 4:**
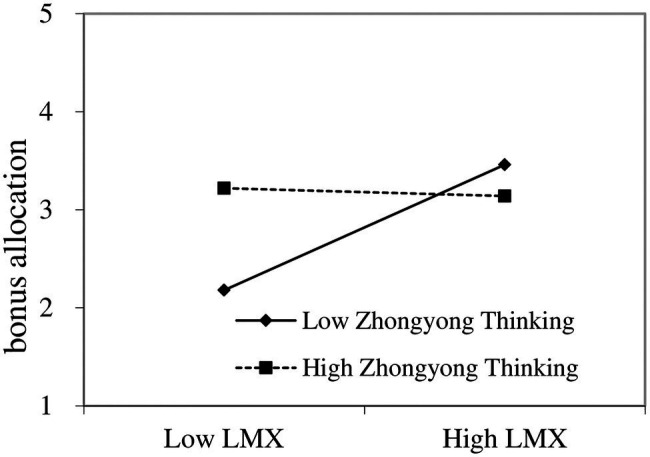
Moderating role of ZYT in the relationship between LMX and bonus allocation.

We depicted the interaction between LMX and ZYT on the supervisors’ HR decisions graphically. Following the study by Aiken and West (1991), we plotted the relationships between LMX and chance of promotion at 1 SD above (high ZYT, 3.75–5) and 1 SD below (low ZYT, 1–2.55) the mean of ZYT. The plot in Figure 3 shows that when supervisors had lower (rather than higher) levels of ZYT, LMX was more positively associated with chance of promotion. The simple slope test confirmed our findings that LMX is positively and significantly related to chance of promotion for low-ZYT supervisors, but the relationship is not significant among high-ZYT supervisors, i.e., H3b is supported. The plot in Figure 4 shows that when supervisors had lower (rather than higher) levels of ZYT, LMX was more positively associated with bonus allocation. The simple slope test confirmed our findings that LMX is positively and significantly related to bonus allocation for low-ZYT supervisors, but the relationship is not significant among high-ZYT supervisors, i.e., H3c is supported.

We used the PROCESS macro Model 14 for SPSS (Hayes, 2013) to test H4a, H4b, and H4c and the results are provided in Table 3. The results demonstrated that the interactive effects of supervisors’ ZYT and LMX on both chance of promotion (*β* = −0.10, *p* < 0.05) and bonus allocation (*β* = −0.11, *p* < 0.01) were negative and significant, and the interactive effect of supervisors’ ZYT and LMX on participation in the decision-making (*β* = −0.00, *p* > 0.05) was insignificant. Additionally, the dependent variable models showed that LMX was positively related to chance of promotion (*β* = 0.96, *p* < 0.01) and bonus allocation (*β* = 0.46, *p* < 0.01). Finally, we analyzed the conditional indirect effect of relationship mediated by LMX. Using 95% CIs, these results revealed that the relationship was significant at the lower levels of ZYT (i.e., −1 SD below the mean) for both chance of promotion [CI = (0.15, 0.56)] and bonus allocation [CI = (0.08, 0.29)]. However, at the higher levels of ZYT (i.e., +1 SD above the mean), the results were not significant for either chance of promotion [CI = (−0.15, 0.20)] or bonus allocation [CI = (−0.03, 0.11)]. Taken together, H4b and H4c were supported, but H4a was not supported. In other words, the impact of upward ingratiation outside the workplace is transmitted to chance of promotion and bonus allocation *via* LMX such that the increases in ZYT reduce the positive consequences of LMX.

**Table 3:** Tests of moderated mediation.

	Mediator model		Dependent variable models					
	LMX		PDM		CP		BA	
	*β*	*SE*	*β*	*SE*	*β*	*SE*	*β*	*SE*
Constant	2.39	0.38	2.45^*^	1.22^*^	−0.73	1.14	−1.09	1.07
IOW	0.37^**^	0.06^**^	0.12	0.07	0.19^**^	0.06^**^	0.29^**^	0.10^**^
LMX			0.01^*^	0.36^*^	0.96^**^	0.34^**^	0.46^**^	0.22^**^
ZYT			−0.05	0.36	0.69^*^	0.34^*^	0.34^*^	0.22^*^
LMX × ZYT			−0.00	0.05	−0.10^*^	0.05^*^	−0.11^**^	0.05^**^
*R*^2^	0.26^**^		0.27^**^		0.25^**^		0.27^**^	
Mediator: LMX			Estimate (SE)	CI	Estimate (SE)	CI	Estimate (SE)	CI
−1 SD ZYT			−0.01 (0.05)	[−0.10,0.09]	0.36 (0.10)	[0.15,0.56]	0.17 (0.05)	[0.08,0.29]
Mean ZYT			−0.01 (0.03)	[−0.06,0.05]	0.12 (0.07)	[−0.01,0.26]	0.08 (0.03)	[0.03,0.14]
+1 SD ZYT			−0.01 (0.04)	[−0.08,0.06]	0.03 (0.09)	[−0.15,0.20]	0.04 (0.03)	(−0.03,0.11)

*n* = 252. Bootstrap sample size = 5,000; 95% CI; *β*, unstandardized regression coefficient. LMX, leader-member exchange; ZYT, Zhongyong thinking; IIW, ingratiation in the workplace; IOW, ingratiation outside the workplace; PDM, participating in decision-making; CP, chance of promotion; BA, bonus allocation.

* *p* < 0.05;

** *p* ≤ 0.01.

## Discussion and Conclusion

Based on the theory of impression management, we examined the mechanisms through which upward ingratiation outside the workplace is related to the supervisors’ HR decisions, and the roles of LMX and ZYT in this influence. Through a questionnaire survey, this research conducted an empirical study based on 252 responses of supervisor-subordinate dyads in China. We found that supervisors hold a more favorable view about ingratiation outside the workplace. In addition, when controlling for ingratiation in the workplace, the results revealed that ingratiation outside the workplace was positively related to the supervisor’s chance of promotion and bonus allocation decisions, and that the relationship between ingratiation outside the workplace and the supervisor’s chance of promotion and bonus allocation decisions was partially mediated by LMX. In addition, ZYT moderates the relationship between ingratiation outside the workplace and supervisor’s chance of promotion and bonus allocation decisions. The moderated mediation results demonstrated that when supervisors use ZYT to consider the people and things around, it serves as a buffer that reduces the positive impact of ingratiation outside the workplace on both supervisors’ chance of promotion and bonus allocation decisions. However, the influence of ingratiation outside the workplace on participation in the decision-making is not significant. The reason may be that for improving the quality of decision-making and the long-term development of enterprise, the supervisor would decide which subordinate is awarded to participate in the decision-making on the foundation of the judgment on their talent as opposed to LMX.

### Contributions, Limitations, and Future Research

#### Theoretical Contribution

1. There are a lot of empirical research showing that subordinates’ upward ingratiation has a significant influence on HR decisions of supervisors. However, few studies have sought to investigate the underlying theoretical mechanisms of the relationship between the ingratiation behavior outside the workplace and HR decisions of supervisors. Earlier, the research began to explore the relationship between ingratiation and career success (Aryee et al., 1996). However, it still remains unclear how subordinates’ ingratiation outside the workplace affects their career success. Foreign studies basically ignore the implementation of the ingratiation behavior outside the workplace (Liu et al., 2015). On the basis of verifying the necessity of differentiating between ingratiation in the workplace and ingratiation outside the workplace, this study explored how ingratiation outside the workplace influences the career outcomes of employees in the workplace, which can be an important contribution to the existing ingratiation research in the West. This study also confirmed that the ingratiation outside of the workplace is more effective than the ingratiation in the workplace in predicting the career development of subordinates. At the same time, it also provided an explanatory perspective for the previous studies on the inconsistent conclusions of ingratiation in the workplace, so that researchers can shift their attention to ingratiation outside the workplace.
2. The LMX is shown to be a critical mediator mechanism which better helps to comprehend how and why perceived ingratiation outside the workplace is beneficial to the subordinate. This corresponds to the growing evidence that the relationship quality seems to influence career development outcomes. For example, the study by Alexander and Wilkins (1982) revealed that the relationship quality was a stronger predictor of job performance ratings than objective performance measures. Duarte et al. (1994) and Wayne et al. (1997) found similar results using LMX as a relational measure. This indicates that supervisors may behave more beneficially to subordinates with whom they share a better quality relationship, possibly motivated by some forms of social reciprocity. This study extended the model of Liu et al. (2015) by including ingratiation outside the workplace and using LMX to explain how and why this deviation affects the HR decisions of supervisors.
3. This study examined an important indigenous concept, ZYT, which is a relatively important psychological variable derived from the traditional Chinese culture. Less attention has been paid to ZYT of supervisor in the existing studies (Chen et al., 2010). This study improved the understanding of ZYT with a Chinese indigenous research perspective by examining its moderating role in the relationship between ingratiation outside the workplace and supervisor’s HR decisions. ZYT helps people understand the traditional Chinese Confucian culture (Yang, 2009). ZYT includes not only the identification of the ingratiation behavior, but also the attitude toward the ingratiation behavior itself. It is important to test the moderating role of supervisors’ ZYT because, as previously discussed, the existing research has found mixed results regarding the impact of ingratiation on supervisor’s HR decisions. Without considering the moderating role of supervisor’s ZYT, we probably could not fully test the impression management theory and effectively make a conclusion on how the ingratiation benefits or harms to subordinate’s career. Studying the validity of subordinates’ ingratiation outside the workplace behavior from the perspective of supervisors’ ZYT will play a certain role in highlighting ZYT and previous research on the ingratiation behavior. Furthermore, as a Confucian code of conduct, ZYT contains rich management connotation (Chen et al., 2017). This study was conducive to our understanding of how easterners speak (Qu et al., 2018), the integration of indigenous psychology with modern management and economics research, and the integration of Eastern and Western cultural management research.

#### Practical Implications

1. Subordinates should make sure that the ingratiation behavior is appropriate in particular situation, and they should consciously improve their exchanges with supervisors to transit from an “outsider” to “insider.” The results indicate that in East Asia, where many personal friendships are developed outside the workplace, the ingratiation is a good interpersonal strategy, so it is crucial to carefully choose the circumstances. It is only when supervisors and subordinates produce high LMX that supervisors would make a positive managerial decision based on interpersonal networks and ZYT.
2. For the supervisors, when confronting with ingratiation from subordinates, they had better to keep balance (Zhongyong). In the Chinese culture, when supervisors deal with their subordinates, they adopt two principles, namely, “fairness” and “humanity” (Aryee and Chen, 2006; Westphal and Bednar, 2008; Stanley et al., 2018). Whether supervisors are guided by the Law of Fairness or the Law of Human Relations, their behaviors seek to achieve the goal of harmony and satisfaction (Ling et al., 2019). ZYT helps supervisors confronted with the ingratiation of subordinates make decisions based on subordinates’ talents rather than LMX.

Foreign employees and managers who work in China should adapt to the cultural environment in China, despite that the ingratiation strategies are less frequent in Western organization, and the inherent “interest” is less than in the Chinese organization characterized as a strong power imbalance (Gutierrez et al., 2012; Barkema et al., 2015). The ingratiation in eastern culture has already infiltrated the private sphere. Foreign employees or managers should treat this type of “ingratiation outside the workplace” properly and apply it skillfully.

#### Limitations and Future Research

There are some limitations in this study. The samples applied are taken from traditional production-oriented enterprises. This approach could effectively control the influence of industry and enterprise-related factors and improve the internal validity of this study, while it limits the external validity of this study to some extent. Follow-up studies should be carried out with more extensive industry and regional surveys to verify the mechanism that drives the ingratiation behavior outside the workplace. At the same time, this study matches the data of supervisors and subordinates at different time points, which helps eliminate the common method deviation, but still involves some subjective components. In future, a longitudinal study should be carried out with the experimental method in order to further improve the accuracy of the research results.

In addition, a number of contextual factors may also limit the universality of our results. We conducted our study in China. Ralston et al. (2005) and Botero et al. (2012) found that culture had a significant influence on both the choice and the effect of upward ingratiation. China is categorized as both collectivist and possessing a high power distance (Zhang and Begley, 2011). Liu et al. (2015) believed that the Chinese ingratiation behavior is quite different from the Western ingratiation behavior. In the Western organization, although it is common that subordinates ingratiate their supervisors, their frequency and internal “interests” are less than those in the east with the high power distance. Therefore, future research should intend to inspect the mediating effects of ingratiation outside the workplace on supervisors’ HR decisions in other cultural contexts, especially those characterized as having low power distance/individualism. Hopefully, there can be more mediation mechanisms to be proposed in the research of the relationship between them.

Apart from that, all the participants involved in this research were from China, and the study on the cultural boundary condition was conducted using ZYT as a moderator. Therefore, the serviceability of the model with cultural characteristics suggested in this study needs to be further tested. The comparative study of different cultures between Eastern and Western countries and the feasibility of ZYT in other cultural contexts provide opportunities for future research.

## Data Availability Statement

The raw data supporting the conclusions of this article will be made available by the authors, without undue reservation.

## Ethics Statement

Ethical review and approval was not required for this study on human participants in accordance with the local legislation and institutional requirements. The patients/participants provided their written informed consent to participate in this study.

## Author Contributions

HS and HG participated in the design, data collection, drafting of the early version, and revision of the manuscript. KW participated in the data collection and analysis, drafting of the early version, and revision of the manuscript. LS and LW participated in the design and revision of the manuscript. All authors contributed to the article and approved the submitted version.

### Conflict of Interest

The authors declare that the research was conducted in the absence of any commercial or financial relationships that could be construed as a potential conflict of interest.
